# The utility of Insulin Like Growth Factor Binding Proteins (IGFBPs-1, 2, 3) with genes expression in resistance to Imatinib and Nilotinib in chronic myeloid leukemia: a pilot study from Delta Egypt

**DOI:** 10.4314/ahs.v24i3.26

**Published:** 2024-09

**Authors:** Nadia El Menshawy, Mohamed Sabry El-Ghonemy, Shaimaa El-Ashwah, Heidi Elkerdawy, Ramy Abbas, Mostafa Abdelhakiem, Maha Saif, Ahmed EL-Sebaie

**Affiliations:** 1 Hematology Unit, Clinical Pathology Department, Faculty of Medicine, Mansoura University, Mansoura, Egypt; 2 Clinical hematology unit, oncology center, Faculty of Medicine, Mansoura university, Egypt; 3 Clinical Hematology, Internal Medicine department, Faculty of Medicine, Port Said University, Port Said; 4 Medical oncology unit, Oncology Center, Faculty of Medicine, Mansoura university, Mansoura, Egypt

**Keywords:** IGFBP-1-3, Gene expression, CML, Imatinib, Nilotinib

## Abstract

**Introduction:**

Resistance to tyrosine kinase inhibitors (TKIs) is an obstacle facing CML patients in spite of the high cure rate. In this context, a study association between IGFBP (1, 2, 3) genes expression and their proteins in CML with the response to TKI has been implicated.

**Patients and methods:**

115 newly diagnosed CML in chronic phase (CP) followed up over 12 months under TKI. 116 apparently healthy individuals were used as a control. RT-qPCR amplification was used for detecting IGFBPs genes expression, and ELISA technique was used for measuring serum IGFBPs.

**Results:**

IGFBP-1 and IGFBP-3 genes expression, as well as their serum levels, were significantly higher in CML patients, whereas IGFBP-2 gene expression was not. Interestingly, IGFBP-1 gene expression and IGFBP-1 serum levels were significantly higher in resistant patients compared to responder patients. However, the expression of IGFBP-2, 3 genes and their serum were insignificant.

**Conclusion:**

IGFBP-1 gene expression and its serum were significantly correlated with resistance. It is currently recommended that IGF-receptor inhibitors be developed and utilized. We are hoping to optimize the cure rate for CML treated with TKIs.

## Introduction

Chronic myeloid leukemia (CML) is a clonal myeloproliferative disorder that originates from a single pluripotent hemopoietic stem cell in which the myeloid lineage cells undergo inappropriate clonal expansion due to a molecular lesion^1^.

The incidence of this disorder represents about 1-2 cases / 100,000 adults in developed countries. It accounts for nearly 15% of newly diagnosed cases of leukemia in adult patients, with a slight male predominance over females. Most patients (around 95%) are diagnosed with CML in the chronic phase (CML-CP) 2.

CML is characterized by the t(9;22) (q34;q11) balanced reciprocal translocation of the Philadelphia (Ph) chromosome, which leads to the generation of the BCR::ABL oncogenic fusion gene encoding the chimeric BCR::ABL protein with constitutive kinase activity 3. Since the introduction of Imatinib IM in 2001 and TKI that targets BCR::ABL, the annual mortality rate in CML has dropped from 10% -20% to 1%-2%. However, TKI resistance, including BCR:: ABL-dependent and independent resistance, is a major problem in TKIs-based CML treatment 4. Insulin and insulin-like growth factors (IGFs) are well known as key regulators of energy metabolism and growth development. These factors and the signal transduction network have important roles in proliferation and protection from apoptosis in neoplasia. Many clinical and laboratory research methods are being used to investigate novel cancer prevention and treatment strategies related to insulin and IGFs signaling 5.

Accumulating evidence demonstrates that the IGF axis not only promotes tumorigenesis but also confers resistance to standard treatment as imatinib (IM) based therapy in CML. IGFs are members of a ligand family that is comprised of A and B chains linked via two disulfide bonds with a third disulfide linkage within the A chain. The two IGF ligands, IGFs-1 and 2, display 67% identity to each other and a high degree (45–52%) of sequence homology with the A and B chains of insulin, but differ due to retention of the bridging C-domain and a C-terminal D-domain extension 6.

The functions of IGFs are mediated through association with the cell surface receptor tyrosine kinases (RTKs) type 1 called IGF receptor (IGF-1R) and insulin receptor (INSR) 7. There are several intrinsic mechanisms in place to regulate IGF activity. The majority of circulating IGF-1/2 is present in high affinity with IGF binding proteins (IGFBPs 1-6) as inactive complexes 8. IGFBPs therefore prolong the circulating half-life of IGFs, although they can undergo proteolytic cleavage to release free IGFs. It is recognized that IGFBPs have more complex functions that can promote IGF bioactivity, and they also have IGF-independent functions 9.

Aberrant IGF signaling with genetic abnormalities and/or chromosomal alterations can result in deregulated expression of IGF ligands, IGF-1R, and IGFBPs. These changes can occur as primary driver events that contribute to cancer growth and tumor progression^10^-^11^ as melanoma 12, bladder cancer 13, gastrointestinal tumors 14, and hematological malignancies 15.

This study aimed to determine the correlation between serum levels of Insulin like growth factor binding proteins (IGFBP) types 1, 2, 3 in chronic myeloid leukemia patients and their gene's expression with the response to TKIs therapy.

## Patients and methods

**Figure 1:**
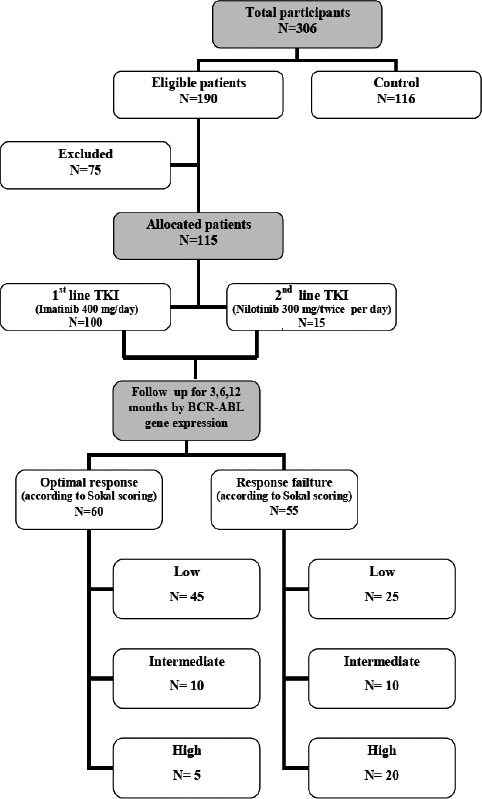
Flow diagram of studied CML patients

After approval of the Local Ethics Committee of Mansoura University, Faculty of Medicine, with an approval number (R.22.12.1997) and obtaining written informed consent from all patients, this study was conducted on 115 newly diagnosed CML patients in chronic phase (CP) at the Oncology Center of Mansoura University (75 males and 40 females), they were stratified by Sokal risk score, with 100 patients receiving imatinib 400 mg/day and 15 patients receiving nilotinib 300 mg twice daily, Fig. (1). The monitoring milestones of BCR::ABL1 transcript levels by the International Scale at 3, 6, and 12 months. Table (1) determined whether the current treatment should be continued (optimal response), changed (failure/resistance/intolerance) 16. They were followed up over 12 months in the period from May 2020 to July 2022. In addition, 116 apparently healthy individuals were subjected to the control group.

**Table 1:** Milestones for treating CML expressed as BCR::ABL1 on the International Scale

	Optimal	Warning	Failure
Baseline	NA	High-risk ACA, high-risk ELTS score	NA
3 months	=10%	>10%	>10% if confirmed within 1–3 months
6 months	=1%	>1–10%	>10%
12 months	=0.1%	>0.1–1%	>1%
Any time	=0.1%	>0.1–1%, loss of =0.1% (MMR)^a^	>1%, resistance mutations, high-risk ACA

For patients aiming at TFR, the optimal response (at any time) is BCR::ABL1 = 0.01% (MR4).

A change of treatment may be considered if MMR is not reached by 36–48 months.

NA: not applicable, ACA: additional chromosome abnormalities in Ph+ cells, ELTS: EUTOS long term survival score,

MMR: major molecular remission, TFR: treatment free remission.

a Loss of MMR (BCR::ABL1 > 0.1%) indicates failure after TFR

The diagnosis of CML was performed in International Canadian accreditation hematological laboratory with molecular and cytogenetic units, based on criteria established by the World Health Organization 17.

Five mililitres of venous blood were withdrawn from the patients at the time of diagnosis, as well as from the control group. The samples were divided into 2 tubes, one containing EDTA (ethylene diamine tetra acetic acid) and another plain tube where serum was separated.

Total RNA was extracted and followed by RT-qPCR amplification for detection of the IGFBP-1, 2, 3 genes expression.

The Power SYBR® Green PCR Master Mix (Applied Biosystems; Thermo Fisher Scientific, Inc.) was used for RT qPCR and the PCR primers were: (IGFBP-1; Forward: 5′ CACAGGGTATGGCTC 3′, IGFBP-1 Reverse: 5′ CTTCTGGGTCTTGGG 3′), (IGFBP-2; Forward: 5′ CGATGCTGGTGCTTCTCA 3′, IGFBP-2 Reverse: 5′ GGGGTCTTGGGTGGG 3′) and (IGFBP-3; Forward: 5′ CTCTCCCAGGCTACACCA 3′, IGFBP-2 Reverse: 5′ GAAGTCTGGGTGCTGTGC 3′)

The mRNA levels were normalized to GAPDH. The changes in mRNA expression levels were measured using the comparative Cq method, as follows: Fold change=2 ΔΔCq. The PCR conditions for all genes were as follows: Initial activation was at 95°C for 30 sec, followed by 40 cycles at 95°C for 3 sec and at 60°C for 30 sec.

Serum samples were used to detect the levels of Insulin like growth factor binding proteins (types; 1, 2 and 3) by the ELISA technique. IGFBP-1 (Catalog No; MBS9425011, IGFBP-2 (Picokine Elisa Kit, Catalog No; MBS177374, and IGFBP-3 (Catalog No; MBS732160) were all purchased from (My BioSource, CA, USA).

The patients sample size was calculated by Stata Corp. 2021. Stata Statistical Software: Release 17. College Station, TX: Stata Corp LLC., and published study by Ren et al., 2020; number of groups is 2, expected effect size (d=0.4), using the t test model: difference between two independent means. The required minimal sample size is 100 subjects per group (total 200 subjects) using α error 5% and a power of 80%. To increase the power and compensate for lost follow-up, 115 CML cases and 116 controls were recruited for the current study 18.

**Statistical Methods:** The collected data was analyzed using the Statistical Package for Social Science (IBM Corp. Released 2017. IBM SPSS Statistics for Windows, Version 25.0. Armonk, NY: IBM Corp.). Student t-test was used to assess the statistical significance of the difference between two study group means. Mann-Whitney Test (U test) was used to assess the statistical significance of the difference in a non-parametric variable between the two study groups. Chi-Square test was used to examine the relationship between two qualitative variables. The ROC curve (receiver operating characteristic) provides a useful way to evaluate the sensitivity and specificity for quantitative diagnostic measures that categorize cases into one of two groups. The optimum cut off point was defined as that which maximized the AUC value. AUC is that a test with an area greater than 0.9 has high accuracy, while 0.7–0.9 indicates moderate accuracy, 0.5–0.7, low accuracy and 0.5 a chance result. Logistic regression analysis was used to identify risk predictors. All reported p values were two-tailed and p< 0.05 was significant 19.

## Results

The present study was conducted on 115 CML patients, whose mean age was 48.6 years. They were 75 males (65.2%) and 40 females (34.8%). In addition, 116 healthy individuals of matched age and gender were served as the control group. Most of the studied cases had a Sokal score of low risk (60.9%). One hundred patients (87%) received imatinib, while 13% received nilotinib. Median BCR::ABL at 3, 6 and 12 months was 0.3, 0 and 0, respectively. About half of cases (52.2%) were shifted to 2nd generation TKI; this was attributed to failure in 91.7% and toxicity in 8.3%. These features were shown in Table (2).

**Table 2:** Clinical and Laboratory features of patients with CML

		CML	
		N=115	
**Gender**	**Male**	**N, %**	**75 (65.2%)**
	**Female**	**N, %**	**40 (34.8%)**
**WBC (x10^9^/L)**	**Median (range)**	**109.9**	**(28.5-605)**
**Hb (gm/dl)**	**Median (range)**	**10.9**	**(8.98-13.6)**
**Platelets (x10^9^/L)**	**Median (range)**	**264**	**(115-912)**
**LDH (IU/L)**	**Median (range)**	**891**	**(130-6770)**
0	**N, %**	**85**	**73.9%**
1	**N, %**	**5**	**4.3%**
2	**N, %**	**25**	**21.7%**
Low	**N, %**	**70**	**60.9%**
Intermediate	**N, %**	**20**	**17.4%**
High	**N, %**	**25**	**21.7%**
Imatinib	**N, %**	**100**	**87.0%**
Nilotinib	**N, %**	**15**	**13.0%**
3-month BCR	**Median (range)**	**0.3**	**(0.083-22)**
ABL	**Median (range)**	**0**	**(0-6.6)**
6-month BCR	**Median (range)**	**0**	**(0-6.5)**
ABL			
12-month BCR			
ABL			
**Shift**	**N, %**	**60**	**52.2%**

Abbreviation: WBC: White blood cells, Hb; Hemoglobin, LDH; Lactic dehydrogenase. BCR ABL; breakpoint cluster region Abelson gene.

**Figure 2:**
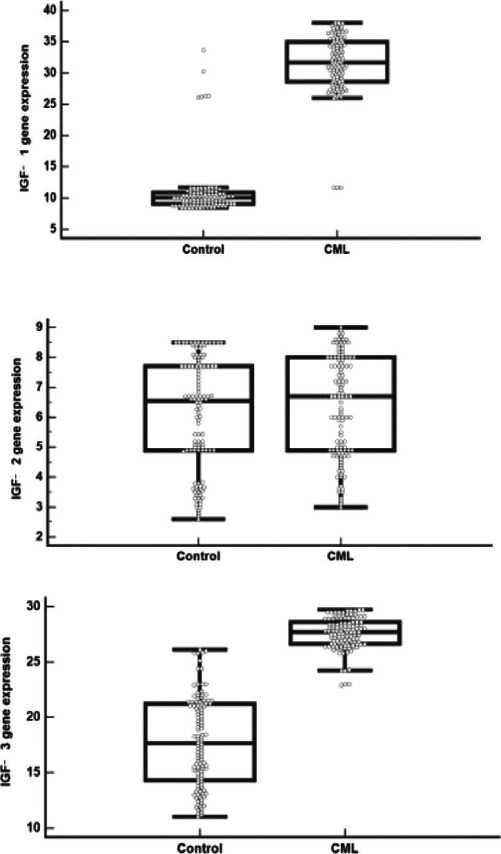
IGFBP (A)1, (B) 2, (C) 3 gene expression levels in CML and control groups

**Figure 3:**
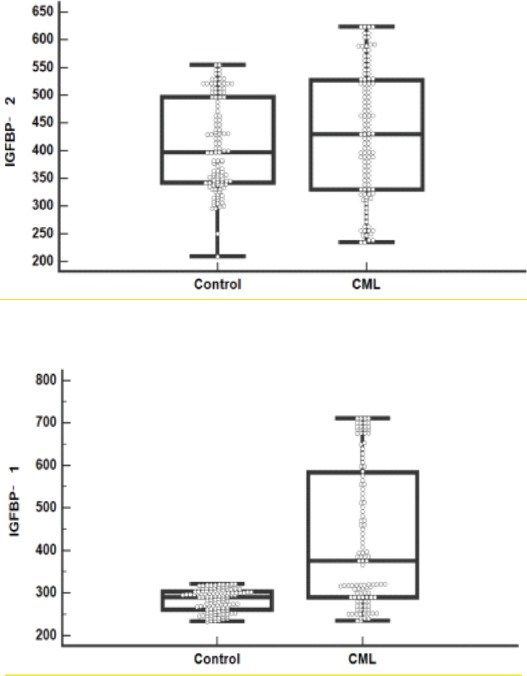
IGFBP (A)1, (B) 2, (C) 3 levels in CML and control groups

IGFBP 1, IGFBP 3 genes expression and IGFBP 1, IGFBP 3 levels were significantly higher in CML patients compared to controls (p< 0.001 for each), while IGFBP 2 gene expression and IGFBP 2 level did not differ significantly between both groups (p= 0.264 and 0.149, respectively) that described in Table (3) and Fig. (2,3).

**Table 3:** Comparison of d IGFBP-1,2,3 genes expression and IGFBP-1,2,3 among studied groups

		Control		CML		P
		N=116		N=115		
IGFBP-1 gene expression	Median (range)	10.1	(8.4-33.7)	31.7	(11.7-38)	< 0.001
IGFBP-2 gene expression	Median (range)	6.6	(2.6-8.5)	6.7	(3-9)	0.264
IGFBP-3 gene expression	Median (range)	17.7	(11-26.1)	27.7	(22.9-29.7)	< 0.001
IGFBP-1 (ng/ml)	Median (range)	290	(233-321)	376	(234-711)	< 0.001
IGFBP-2 (ng/ml)	Median (range)	396	(209-554)	430	(234-623)	0.149
IGFBP-3 (ng/ml)	Median (range)	364	(299-433)	458	(244-634)	< 0.001

On comparing the TKI resistant CML cases to sensitive cases as regards studied parameters, it was found that resistant CML cases were associated with a significant increase in WBC (median= 358 ×109/L), Platelets (median= 388 × 109/L) and LDH (median= 2103 IU/L) levels in resistant cases compared to sensitive cases (p< 0.001 for each), as shown in Table (4).

**Table 4:** Comparison between sensitive and resistant CML cases regarding studied parameters

			Optimal response		Response failure		P
			N=60		N=55		
**Age (years)**		**Mean ± SD**	49.50 ± 9.229		47.64 ± 6.544		0.218
**Gender**	**Male**	**N, %**	35	58.3%	40	72.7%	0.105
	**Female**	**N, %**	25	41.7%	15	27.3%	
**WBC (x109/L)**		**Median (range)**	55.3	(28.5-109.9)	358	(69.93-605)	< 0.001
**HB (gm/dl)**		**Median (range)**	10.9	(8.98-13.6)	10.9	(9-13.6)	0.118
**Platelets (x109/L)**		**Median (range)**	233	(115-374)	388	(115-912)	< 0.001
**Creatinine (mg/dl)**		**Median (range)**	1.1	(0.74-7.8)	0.9	(0.6-1.3)	0.365
**LDH (IU/L)**		**Median (range)**	433	(130-661)	2103	(661-6770)	< 0.001
**N, %**			40	66.7%	45	81.8%	0.018
**N, %**			5	8.3%	0	0.0%	
**N, %**			15	25.0%	10	18.2%	
**Sokal Score**	**Low**	**N, %**	45	75%	25	45.4%	0.018
	**Intermediate**	**N, %**	10	16.7%	10	18.2%	
	**High**	**N, %**	5	8.3%	20	36.4%	
**IGFBP-1 gene expression**		**Median (range)**	28.8	(11.7-33.3)	35.1	(30.9-38)	0.001
**IGFBP-2 gene expression**		**Median (range)**	6.7	(3-8.8)	6.3	(4.7-9)	0.165
**IGFBP-3 gene expression**		**Median (range)**	27.6	(22.9-29.7)	27.8	(23-29.7)	0.574
**IGFBP-1 (ng/ml)**		**Median (range)**	290	(234-711)	586	(365-711)	0.001
**IGFBP-2 (ng/ml)**		**Median (range)**	426	(234-623)	430	(234-623)	0.726
**IGFBP-3 (ng/ml)**		**Median (range)**	455	(244-623)	481	(299-634)	0.583

**Figure 4:**
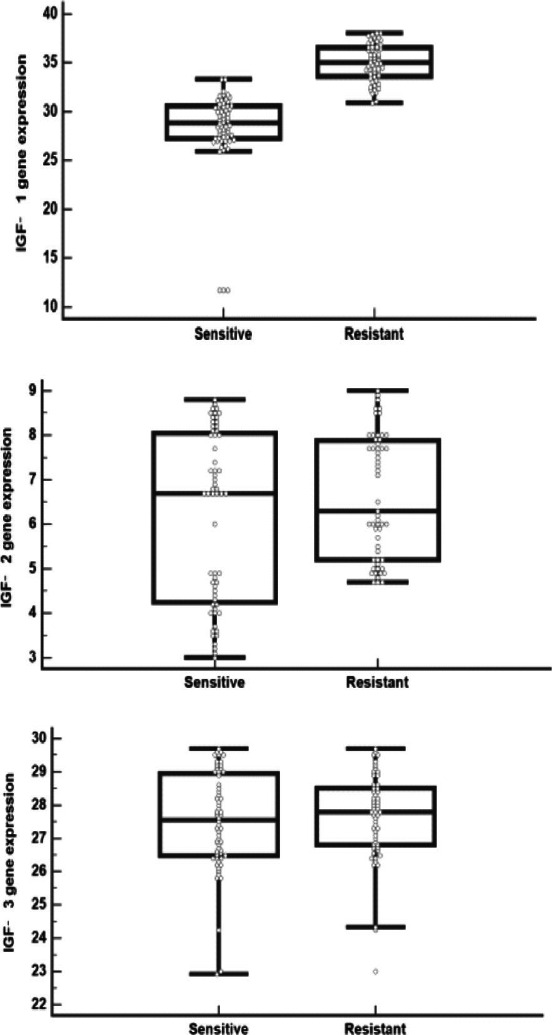
IGFBP (A)1, (B) 2, (C) 3 gene expression levels in sensitive and resistant CML cases

**Figure 5:**
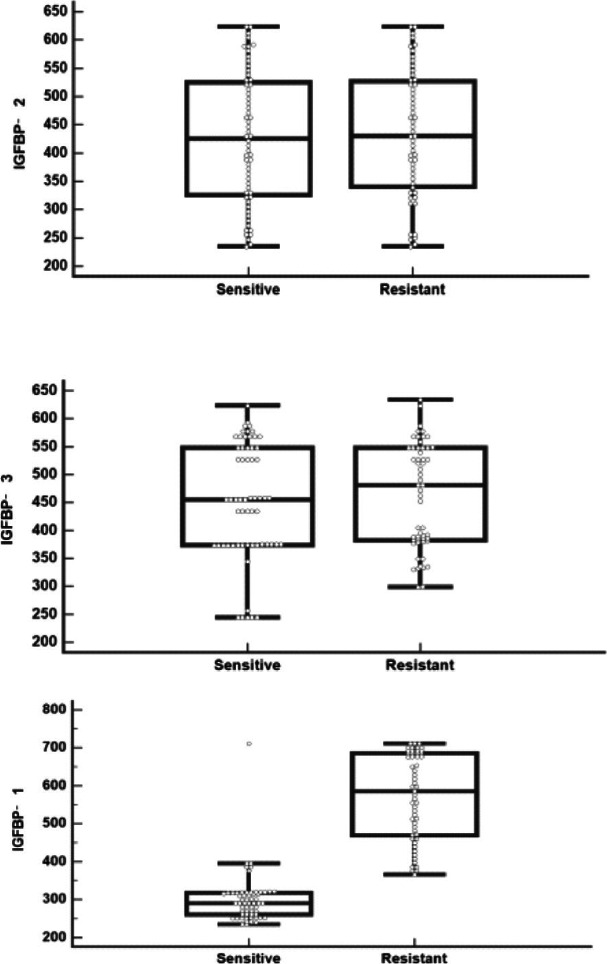
IGFBP (A)1, (B) 2, (C) 3 levels in sensitive and resistant CML cases

IGFBP 1 gene expression and IGFBP 1 level were significantly higher in resistant cases versus sensitive cases (p< 0.001), while insignificant differences of IGFBP 2, 3 genes expression (p= 0.165 and 0.574, respectively) and of IGFBP 2, 3 levels (p= 0.726 and 0.583, respectively) between both subgroups were observed, as shown in Table (4) and Fig. (4,5).

**Figure 6:**
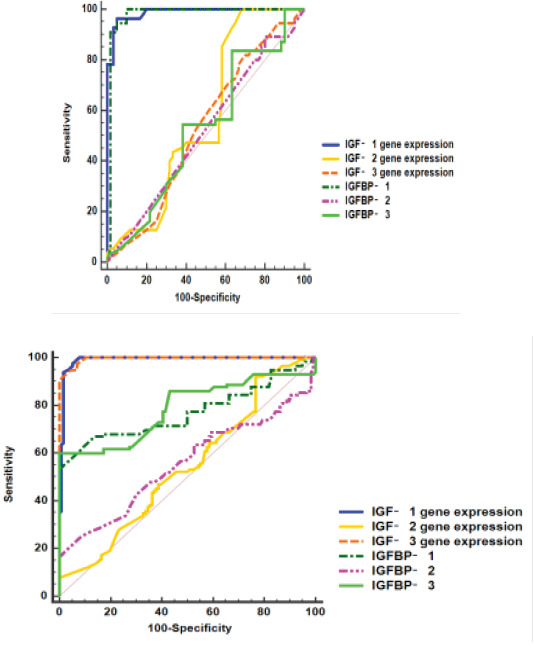
ROC curves of IGE1,2,3, IGFBP 1,2,3 for discrimination between (A) CML and controls, (B) sensitive and resistant CML cases

A ROC curve was conducted for discrimination between CML and controls. IGFBP-1, 3 genes expression showed high accuracy (AUCs= 0.989 and 0.995, respectively); also, IGFBP-1, 3 levels showed moderate accuracy (AUCs= 0.77 and 0.78, respectively), while IGFBP-2 gene expression and IGFBP-2 level showed low accuracy (AUCs= 0.542 and 0.555, respectively), as shown in Table (5) and Fig (6.A).

**Table 5:** Validity of IGFBP-1, 2, 3 genes expression IGFBP-1, 2, 3 for discrimination between studied groups (control vs CML cases) and (Resistant vs Sensitive cases)

Discrimination between	Variable	AUC	95% CI	Cut off	Sensitivity (%)	Specificity (%)
Control and CML	IGFBP-1 gene expression	0.989	0.976 – 1	11.6	100	92.2
	IGFBP-2 gene expression	0.542	0.468- 0.617	6.6	52.2	53.4
	IGFBP-3 gene expression	0.995	0.990- 1	23	97.4	93.1
As shown in Figure 2A	IGFBP-1 (ng/ml)	0.770	0.706- 0.835	298.4	71.3	63.8
	IGFBP-2 (ng/ml)	0.555	0.479 - 0.631	400	56.5	52.6
	IGFBP-3 (ng/ml)	0.788	0.726- 0.849	372	86.1	56
Sensitive and resistant	IGFBP-1 gene expression	0.987	0.972 -1	31.7	96.4	95
	IGFBP-2 gene expression	0.575	0.467 - 0.683	7	47.3	60
	IGFBP-3 gene expression	0.530	0.424- 0.637	27.7	54.1	51.7
As shown in Figure 2B	IGFBP-1 (ng/ml)	0.978	0.946- 1	385.9	94.5	95
	IGFBP-2 (ng/ml)	0.519	0.413- 0.625	429.7	50.9	50
	IGFBP-3 (ng/ml)	0.530	0.423- 0.636	458	54.5	61.7

AUC, area under ROC curve, CI, confidence interval.

Moreover, a ROC curve was conducted for discrimination between sensitive and resistant CML cases. IGFBP-1 gene expression and IGFBP-1 level showed high accuracy (AUCs= 0.987 and 0.978, respectively), while low accuracy was observed in IGFBP-2, 3 genes expression (AUCs= 0.575 and 0.53, respectively) and in IGFBP-2, 3 levels (AUCs= 0.519 and 0.53, respectively). The cut-off values and performance characteristics were also shown in Table (5) and Fig (6.B).

The logistic regression analysis to identify predictive factors for resistance to TKIs treatment in CML cases was presented in Table (6). In the univariate analysis, several factors were examined individually to assess their association with resistance. Higher WBC, platelets, LDH, IGFBP 1 gene expression and IGFBP 1 level were more likely to develop resistance. However, none of the other factors reached statistical significance, including age, gender, diabetes mellitus (DM), hypertension, phase stage, IGFBP 2 and IGFBP 3 genes expression, IGFBP 2 and IGFBP 3 levels. In the multivariate analysis, significant factors were considered simultaneously to determine their independent association with resistance to treatment. Among these factors, higher LDH, IGFBP 1 gene expression and IGFBP 1 level were found to be significant predictors of resistance to TKIs treatment in studied CML cases.

**Table 6:** Logistic regression analysis for analyzing predictors of resistance to treatment

	Univariable			Multivariable		
	p	OR	95% CI	p	OR	95% CI
**Age (years)**	0.213	0.982	0.954-1.011			
**Gender**	0.105	1.495	0.919-2.433			
**DM**	0.831	1.068	0.584-1.955			
**Hypertension**	0.125	0.477	0.249-1.912			
**WBC (x10^9^/L)**	<0.001*	1.014	1.007-1.020	0.201	1.097	0.952-1.265
**Platelets (x10^9^/L)**	<0.001*	1.003	1.002-1.005	0.293	0.978	0.939-1.019
**LDH (IU/L)**	0.004*	1.002	1.001-1.003	0.030*	1.013	1.001-1.062
**IGF-1 gene expression**	<0.001*	2.660	1.748-4.048	0.024*	1.874	1.669-2.142
**IGF-2 gene expression**	0.130	1.114	0.969-1.280			
**IGF-3 gene expression**	0.637	1.039	0.885-1.221			
**IGFBP-1** (ng/ml)	<0.001*	1.012	1.009-1.016	0.001*	1.027	1.010-1.043
**IGFBP-2** (ng/ml)	0.704	1.000	0.998-1.002			
**IGFBP-3** (ng/ml)	0.542	1.001	0.998-1.003			

OR, odds ratio; CI, confidence interval.

## Discussion

CML is the most common form of chronic myeloproliferative diseases and is characterized by a balanced genetic translocation, t(9;22), involving a fusion of the Abelson gene (ABL1) from chromosome 9q34 with the breakpoint cluster region (BCR) gene on chromosome 22q11. This translocation results in the generation of a BCR:ABL1 fusion oncogene translated into a BCR::ABL1 chimeric protein that possesses an active ABL tyrosine kinase 20-21.

The IGF system plays an essential role in normal growth throughout fetal life and childhood. This system continues to function in adults by regulating normal cellular proliferation and differentiation as it protects against the apoptotic process. However, it can contribute to the development and progression of malignant growth 22.

TKI resistance in CML is a frequent event and causes a major clinical challenge during the journey of the treatment. This resistance could be attributed to a gene mutation in BCR::ABL that happens in 40 - 90% of the study by Yang et al. 23, it may be an epigenetic mechanism in Src family kinase or abnormal expression of tumor drug resistance-associated proteins such as IGF as illustrated in the study by El Fakih et al.^24^.

Our study aimed to determine the correlation between insulin like growth factor binding proteins level in chronic myeloid leukemia patients with their gene's expression and the response to TKI therapy.

This study was performed on 115 newly diagnosed chronic myeloid leukemia patients in the chronic phase (CP) with a mean age of (48.6) years admitted to the Oncology Center of Mansoura University (75 males and 40 females) and followed up over 12 months. Also, 116 apparently healthy individuals were served as a control group.

In this study, it was observed that WBCs, platelets count and LDH level at diagnosis in responder cases were significantly lower than in resistant cases (p< 0.001 for each). Furthermore, responder patients had a low Sokal risk score compared to other groups (p= 0.018). Some studies evaluated the prognostic value of WBC counts at presentation in CML on the patients' response and outcome. During the establishment of Sokal risk scores, WBC counts were prognostic for the univariate analyses but insignificant for the multivariable regressions for survival 25-30.

In the present study, we measured the serum levels of IGFBP-1, 2, 3, also we evaluated the expression of IGFBP-1, 2, 3 genes in patients with CML compared to the control group. We found that IGFBP-1, IGFBP-3 genes expression and IGFBP-1, IGFBP-3 levels were significantly higher in CML patients compared to controls (p< 0.001 for each) while IGFBP-2 gene expression and IGFBP-2 level did not differ significantly between both groups (p= 0.264 and 0.149, respectively). On comparison of IGFBP-1, 2, 3 genes expression and IGFBP-1, 2, 3 levels among sensitive and resistant cases, it was observed that IGFBP-1 gene expression and IGFBP-1 level were significantly higher in resistant cases versus sensitive cases (p< 0.001) while there was an insignificant difference between IGFBP-2, 3 gene expression (p= 0.165 and 0.574, respectively) and IGFBP-2, 3 levels (p= 0.726 and 0.583, respectively) between both subgroups.

The study of Ren et al.^18^ was more or less in agreement with our study; they reported that the IGFBP 1 level was higher in the peripheral blood of patients with drug resistance compared with Imatinib sensitive patients and healthy subjects (p< 0.05), whereas the levels of IGFBP 2 and IGFBP 3 were lower.

IGFBP-1 acts as a negative regulator of BAK-dependent apoptosis, and its expression is involved in the transcriptional and mitochondrial functions of the P53 tumor suppressor proteins. A study by Jiang et al. 31 illustrated that P53 signaling pathway that regulates redox status has an essential role in IM resistance.

Pollak 5 declared that IGF-I and IGF-II bind to the insulin-like growth factor receptors IGF-IR and IGFIIR that enhance cell proliferation. Most functions of IGFs are mediated by the IGF-IR, which activates the Ras, Raf, MAPK signaling pathways, as well as by the phosphatidylinositol 3 kinase (PI3K) pathway. Binding of IGF with receptor induces a conformational change that activates the kinase domain subunit, resulting in autophosphorylation of specific tyrosine residues, leading to receptor activation 32.

Sachdev et al.^33^ illustrated that the IGF-1/IGF-1R signaling pathway plays an essential role in the development and progression of many cancers through enhancing proliferation and inhibition of apoptosis. In many cancers, there were overexpressions of IGF-1R and increased IGF-1R tyrosine kinase activity.

IGF-1 is important in leukemogenesis as it stimulates myeloid and lymphoid cells in culture. IGF-1 promotes cell growth and survival through PI3K/Akt signaling detected in leukemic cells 34.

Beltskiy et al.^35^ illustrated that IGF-1,2 signaling plays a role in learning and memory as well as neuroprotection and can be targeted therapy in Alzheimer disease.

A previous study by Lee and Cohen 36 confirmed that IGFBPs adjust the biological activity of IGF by sequestering IGFs away from IGF-Rs, thereby inhibiting the mitogenic and antiapoptotic activities of IGFs. Among the six IGFBPs, IGFBP-3 is the most plentiful protein in the circulation. However, Mehrian-Shai et al.^37^ documented that overexpression of IGFBPs is associated with increased IGF action, leading to bad adverse effects on cancer prognosis.

Chen et al.^38^ in an experimental study, illustrated that silencing the IGFBP3 gene mediated inhibition of the ERK/mitogen-associated protein kinase pathway in proliferation, apoptosis, autophagy and cell senescence. This could be explained by two possible hypotheses. One hypothesis is that the secretion of these high-affinity IGFBPs increases the concentration of IGF ligands in the tumor microenvironment, which are present in an inactive form and released continuously as bioavailable ligands via the action of IGFBP proteases secreted from neoplastic cells. Another hypothesis supposes that IGFBPs may be involved in the activation of integrin-linked kinase 39-40-41.

Hua et al. 6 reported that the blockade of IGF-IR might counteract the Imatinib resistance of CML cells, and so this therapeutic strategy appears to be effective in the treatment of patients with CML, particularly during the aggressive stages or in cases resistant to Imatinib.

## Conclusion

Despite TKI being the drug of choice in the treatment of CML patients, many cases show treatment failure with a bad prognosis. IGFBP-1 gene expression and IGFBP-1 were significantly higher in resistant cases than in sensitive cases. New selective and specific IGFIR inhibitors are currently indicated to be developed and utilized as personalized medicine to overcome resistance to TKI.
